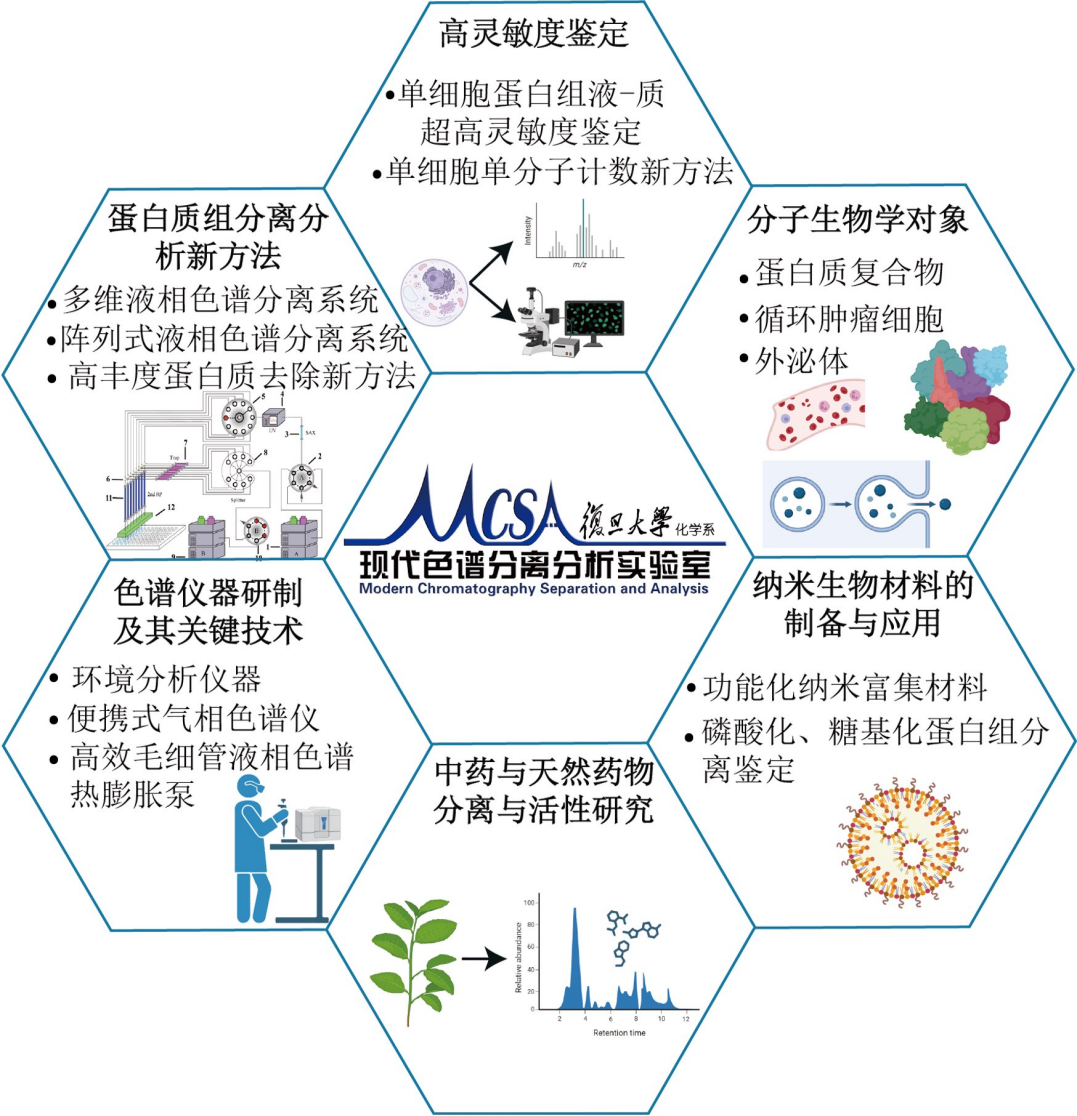# 高效液相色谱-质谱技术在蛋白质组学中的应用

**DOI:** 10.3724/SP.J.1123.2023.11006

**Published:** 2024-07-08

**Authors:** Wei LIU, Lingxiao WENG, Mingxia GAO, Xiangmin ZHANG

**Affiliations:** 1.复旦大学化学系,上海 200438; 1. Department of Chemistry, Fudan University, Shanghai 200438, China; 2.复旦大学生物医学研究院,上海 200032; 2. Institutes of Biomedical Sciences, Fudan University, Shanghai 200032, China

**Keywords:** 高效液相色谱, 反相液相色谱, 亲水相互作用色谱, 疏水作用色谱, 离子交换色谱, 体积排阻色谱, 蛋白质组学, high performance liquid chromatography (HPLC), reversed-phase liquid chromatography (RPLC), hydrophilic interaction liquid chromatography (HILIC), hydrophobic interaction chromatography (HIC), ion exchange chromatography (IEC), size-exclusion chromatography (SEC), proteomics

## Abstract

蛋白质组学研究在生物医学领域发挥了重要作用,然而研究面临的主要难点在于其研究对象的复杂性和多样性。随着质谱技术的快速发展,高效液相色谱-质谱(HPLC-MS)分离分析复杂生物样品已经成为蛋白质组学研究的基础工具。蛋白质组学的研究从肽段分离,延伸到蛋白质和蛋白质复合物的分离,随着分析物的分子质量不断增大,其结构和组成复杂性也持续增加,分子特性也发生改变。面对不同的蛋白质组学研究对象,选择不同的分离模式、分离条件以及固定相参数是进行深度蛋白质组学研究的关键。本文综述了实验室常用的液相色谱分离模式,包括反相色谱(RPLC)、亲水相互作用色谱(HILIC)、疏水相互作用色谱(HIC)、离子交换色谱(IEC)和体积排阻色谱(SEC),以及其不同的组合模式与质谱联用在自下而上(bottom-up)分析、自上而下(top-down)分析、蛋白-蛋白相互作用分析中应用的研究。具体分析了色谱流动相与被分析对象之间的兼容性问题、色谱流动相与质谱兼容性问题,以及多维色谱中不同色谱模式之间流动相的兼容性问题。重点关注存在不兼容问题时研究者所提出的解决方案。此外,本文还评述了HPLC-MS结合样本前处理的方法在外泌体和单细胞蛋白质组学中的应用研究。总之,文章聚焦于近年来HPLC-MS技术在蛋白质组学中的研究进展,旨在为未来蛋白质组学领域的研究提供参考。

## Abstract (en)

Proteomics profiling plays an important role in biomedical studies. Proteomics studies are much more complicated than genome research, mainly because of the complexity and diversity of proteomic samples. High performance liquid chromatography-mass spectrometry (HPLC-MS) is a fundamental tool in proteomics research owing to its high speed, resolution, and sensitivity. Proteomics research targets from the peptides and individual proteins to larger protein complexes, the molecular weight of which gradually increases, leading to sustained increases in structural and compositional complexity and alterations in molecular properties. Therefore, the selection of various separation strategies and stationary-phase parameters is crucial when dealing with the different targets in proteomics research for in-depth proteomics analysis. This article provides an overview of commonly used chromatographic-separation strategies in the laboratory, including reversed-phase liquid chromatography (RPLC), hydrophilic interaction liquid chromatography (HILIC), hydrophobic interaction chromatography (HIC), ion-exchange chromatography (IEC), and size-exclusion chromatography (SEC), as well as their applications and selectivity in the context of various biomacromolecules. At present, no single chromatographic or electrophoretic technology features the peak capacity required to resolve such complex mixtures into individual components. Multidimensional liquid chromatography (MDLC), which combines different orthogonal separation modes with MS, plays an important role in proteomics research. In the MDLC strategy, IEC, together with RPLC, remains the most widely used separation mode in proteomics analysis; other chromatographic methods are also frequently used for peptide/protein fractionation. MDLC technologies and their applications in a variety of proteomics analyses have undergone great development. Two strategies in MDLC separation systems are mainly used in proteomics profiling: the “bottom-up” approach and the “top-down” approach. The “shotgun” method is a typical “bottom-up” strategy that is based on the RPLC or MDLC separation of whole-protein-sample digests coupled with MS; it is an excellent technique for identifying a large number of proteins. “Top-down” analysis is based on the separation of intact proteins and provides their detailed molecular information; thus, this technique may be advantageous for analyzing the post-translational modifications (PTMs) of proteins. In this paper, the “bottom-up” “top-down” and protein-protein interaction (PPI) analyses of proteome samples are briefly reviewed. The diverse combinations of different chromatographic modes used to set up MDLC systems are described, and compatibility issues between mobile phases and analytes, between mobile phases and MS, and between mobile phases in different separation modes in multidimensional chromatography are analyzed. Novel developments in MDLC techniques, such as high-abundance protein depletion and chromatography arrays, are further discussed. In this review, the solutions proposed by researchers when encountering compatibility issues are emphasized. Moreover, the applications of HPLC-MS combined with various sample pretreatment methods in the study of exosomal and single-cell proteomics are examined. During exosome isolation, the combined use of ultracentrifugation and SEC can yield exosomes of higher purity. The use of SEC with ultra-large-pore-size packing materials (200 nm) enables the isolation of exosomal subgroups, and proteomics studies have revealed significant differences in protein composition and function between these subgroups. In the field of single-cell proteomics, researchers have addressed challenges related to reducing sample processing volumes, preventing sample loss, and avoiding contamination during sample preparation. Innovative methods and improvements, such as the utilization of capillaries for sample processing and microchips as platforms to minimize the contact area of the droplets, have been proposed. The integration of these techniques with HPLC-MS shows some progress. In summary, this article focuses on the recent advances in HPLC-MS technology for proteomics analysis and provides a comprehensive reference for future research in the field of proteomics.

色谱法是一项重要且被广泛应用的生物物理技术,可以对复杂的组分进行分离、纯化以实现特定组分的鉴定与定量。高效液相色谱(high performance liquid chromatography, HPLC)由于其卓越的分离效能以及稳定可靠的性能而被广泛应用于生物大分子(如蛋白质复合物、蛋白质、多肽、核酸、多糖)的分离^[1]^。在HPLC分离生物大分子的应用中,根据分子性质和分析要求可采用不同的分离模式:(1)反相液相色谱(reversed-phase liquid chromatography, RPLC)使用非极性固定相和极性流动相,通过疏水相互作用的不同进行分离^[2]^。(2)亲水相互作用色谱(hydrophilic interaction liquid chromatography, HILIC)使用亲水性固定相,其保留机制包括亲水相互作用和二次静电和氢键相互作用的复杂组合^[3]^。(3)疏水相互作用色谱(hydrophobic interaction chromatography, HIC)利用被分离物质与固定相之间弱的疏水相互作用进行分离,固定相填料一般是在亲水化处理的微球表面修饰疏水性基团,流动相一般为pH 6~8的盐溶液^[3]^。(4)离子交换色谱(ion exchange chromatography, IEC)根据分析物组分离子与离子交换剂的亲和力不同来对其进行分离,几乎适用于任何类型的带电分子,包括大分子蛋白质、小分子核苷酸和氨基酸等,其流动相一般为一定浓度梯度的盐溶液,生物兼容性比较好^[4]^。(5)体积排阻色谱(size-exclusion chromatography, SEC)主要依据被分离物质的分子质量大小进行分离^[5]^。为确保准确、高效地分离和分析生物大分子,根据样品中待测物质的分子性质以及复杂程度,选择合适的HPLC分离模式及多种分离模式的联用至关重要。

在后基因组学时代,基于质谱(mass spectrometry, MS)的蛋白质组学技术因其卓越的定量能力,在高通量蛋白质鉴定与定量中发挥着不可或缺的作用^[6]^。目前,高效液相色谱-质谱技术(HPLC-MS)日趋成熟,可以对各种生物样本中成千上万种蛋白质进行规模化鉴定,定量分析逐步实现“深度覆盖”,从而帮助研究人员更深入地了解生物体的各种基础生理过程(例如生长、发育、衰老)以及病理过程(例如炎症、肿瘤)的分子基础,也使我们能够迅速应对未知疾病的挑战。一个典型的案例是,在COVID-19全球大爆发初期,研究人员快速地完成了对COVID-19患者血浆^[7]^、尿液^[8]^、组织样本^[9]^、严重急性呼吸综合征冠状病毒2(SARS-COV-2)感染的宿主细胞^[10]^的蛋白质组学研究。通过对COVID-19患者血浆蛋白质组的研究,研究者发现了感染SARS-CoV-2后引起的关键信号通路的改变,并通过蛋白质组学和代谢组学的数据构建了可以区分COVID-19严重程度的分类器^[7]^。通过对COVID-19患者尿液蛋白质组的研究,研究者揭示了COVID-19感染早期的免疫抑制状态和在重型COVID-19患者中出现的免疫激活状态^[8]^。通过对COVID-19感染死亡的尸检组织样本的蛋白质组学研究^[8]^,研究者发现COVID-19患者肺部的组织蛋白酶L1显著上调。虽然血管紧张素转化酶2 (ACE2)介导SARS-CoV-2进入人体^[11]^,但并未在肺组织发生明显改变。这个结果提示ACE2抑制剂可能不是治疗重症和危重症COVID-19患者的有效疗法^[9]^。通过对SARS-CoV-2感染的宿主细胞的蛋白质组研究确定了受到SARS-CoV-2扰动的宿主细胞信号通路,并且找到了阻止病毒复制的潜在药物^[10]^。这些研究成果为其他领域的科研工作者提供了极大的帮助,使他们能够更深入地了解并应对COVID-19这一全球性卫生危机。

本文对基于HPLC-MS的蛋白质组学技术的研究进展进行了综述,分别介绍了HPLC-MS技术在蛋白质组学自下而上(bottom-up)分析、自上而下(top-down)分析、蛋白-蛋白相互作用分析以及当前热点研究的生物学对象中的应用,其中重点突出了多维色谱分离模式的选择以及分离条件的优化,最后探讨了HPLC-MS技术的应用潜力。

## 1 在自下而上的蛋白质组学中的应用

根据研究对象的不同,蛋白质组学研究策略可分为自下而上的蛋白质组学(bottom-up proteomics, BUP)^[12]^和自上而下的蛋白质组学(top-down proteomics, TDP)^[13]^。BUP分析的主要对象是蛋白质酶解产物,即通过特定蛋白酶将蛋白质酶解为肽段,使用质谱对这些肽段进行检测,通过将肽段碎裂产生的二级质谱谱图与计算机根据蛋白质数据库生成的理论谱图进行比较来实现肽段鉴定^[14]^。这种通过肽段信息去表征蛋白质的策略又称为鸟枪法(shotgun)蛋白组学,这个概念最早由Yates实验室提出^[14,15]^,典型的分离模式是强阳离子交换(strong-cation exchange, SCX)-RPLC^[16]^。相对于直接对蛋白质进行鉴定,该策略的优势如下:(1)经过蛋白酶酶解产生的肽段分子质量大多为500~3000 Da,更容易通过色谱分离;(2)肽段C末端存在赖氨酸或精氨酸残基(使用胰蛋白酶酶解),使得肽段在酸性条件下能够被有效质子化,因此产生的肽段在气相色谱中能够很好地电离和碎裂^[17]^; (3)经过蛋白酶酶解形成的肽段具有可预测的末端和碎裂形式,这有利于蛋白质组学数据的解析^[18]^。这些优势使得鸟枪法蛋白质组学可以普遍地应用于蛋白质分析,进一步推动人类蛋白质组学草图^[19]^以及高精度的人类蛋白质组学图谱^[20]^的完成。

### 1.1 RP-RPLC多维分离策略

RPLC是当前进行肽段分离的首选方法,其固定相通常为C18修饰的硅球,孔径一般为10~12 nm^[21,22]^。RPLC流动相为有机溶剂,不会影响肽段的电离,且可以连接各种离子源,因此,当肽段从色谱柱洗脱后,它们可以直接进入质谱分析。由第一维RPLC使用高pH的流动相、第二维RPLC使用低pH的流动相组成的高/低pH RP-RPLC分离模式以其强大的分离能力在蛋白质组学研究中得到广泛的应用。蛋白质磷酸化是真核生物中最重要的翻译后修饰之一^[23]^, BUP策略通过表征磷酸化肽段来分析蛋白质磷酸化修饰^[24]^,即先对磷酸化肽段进行预富集,然后进行HPLC-MS检测,最后完成数据解析^[25]^。为了提高磷酸化肽段鉴定的深度,Song等^[21]^开发了一种用于磷酸化肽分离的离线二维RP-RPLC方法。该方法通过在第一维的流动相中添加25 mmol/L甲酸铵(pH 7.5)作为高pH RPLC,第二维的分离流动相中添加0.1%甲酸(pH 2.0)作为低pH RPLC,采用间隔合并馏分的方法减少总馏分数量的同时提高质谱检测窗口的利用率,最后磷酸化肽段鉴定数量相比传统高/低pH RP-RPLC提高了30%。此外,在定量蛋白质组分析的研究中,也通常使用高/低pH RP-RPLC对多通道标记的肽段样本进行二维分离,以降低样品复杂性,提高蛋白质组学鉴定覆盖率^[26]^。

在离线二维高/低pH RP-RPLC分离中,通常需要在第一维高pH RPLC分离阶段使用0.1~1 mg的肽段作为初始样品,使用内径为2.1~4.6 mm的色谱柱,以0.1~1.0 mL/min的流速进行分离^[27]^。而第二维低pH RPLC通常是在与高分辨质谱联用的纳升级色谱系统中运行,通常ng级的肽段样本足够用于分析^[28]^。Zurawska等^[29]^利用小内径(300 μm)的色谱柱和低流速(5 μL/min)成功开发了适用于少量肽段(30~60 μg)的高pH RPLC分离系统。在进行低流速分析时,为了解决高pH流动相中可能会出现因盐分析出导致系统压力不稳定和堵塞等问题,研究者测试了9种高pH缓冲液/溶剂系统,最终筛选出20 mmol/L碳酸氢铵(pH 8.5)作为缓冲体系。随着分离条件的不断优化,二维高/低pH RP-RPLC技术路线逐步成熟,成为蛋白质组学研究中不可或缺的研究手段。

### 1.2 HILIC-RPLC多维分离策略

HILIC与RPLC因分离原理不同,二者有更高程度的正交性^[30]^。Roca等^[22]^开发了一种低流速的在线二维LC系统,并将其应用于分离肽段。该系统的第一维为HILIC,色谱柱内径为200 μm,流速为1 μL/min,第二维色谱为RPLC,色谱柱柱内径为100或150 μm,流速为1.2 μL/min。使用小尺寸的色谱柱使得该系统可以对少量样品(1~5 μg肽段)进行二维分离后直接进行质谱鉴定。HILIC需要从高浓度的有机相逐渐过渡到低浓度的有机相。高浓度的有机相可能导致从HILIC洗脱的肽段在RPLC上无法保留。为了解决HILIC和RPLC流动相不兼容的问题,研究者通过一个T型分流器将补充液(含0.1% FA的水相)以9 μL/min流速与来自第一维的馏分混合,从而稀释HILIC洗脱液中的高浓度乙腈。研究者在两个维度之间引入了C18捕集柱,将从HILIC洗脱后被稀释的分析物进行浓缩,然后以1.2 μL/min的流速进行第二维RPLC分离,从而实现了在线二维分离(图1)。与一维RPLC分离相比,在相同的质谱分析时间下该二维系统的峰容量增加了60%^[22]^。

**图1:**
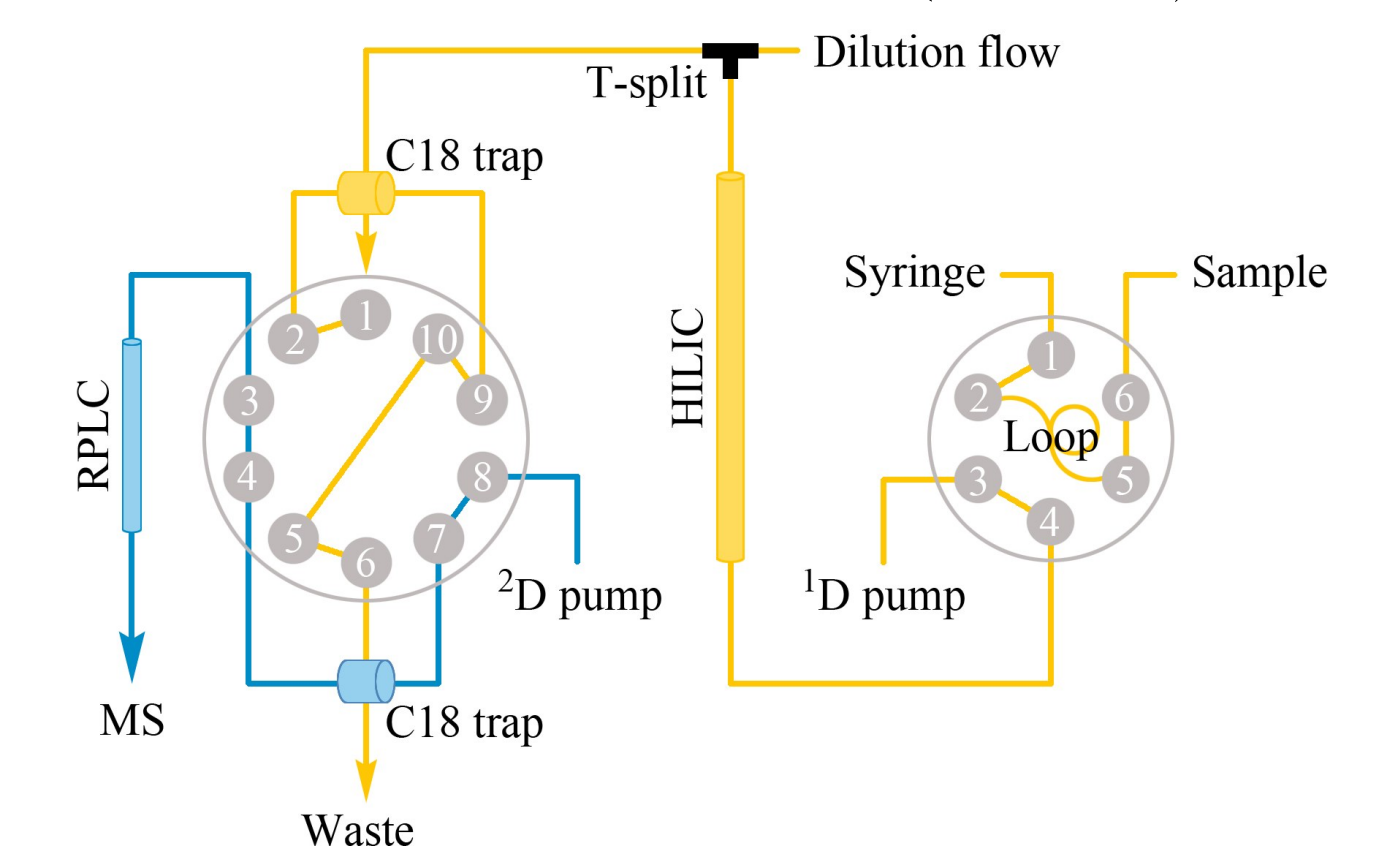
HILIC-RPLC在线二维分离示意图^[22]^

### 1.3 IEC-RPLC多维分离策略

数据非依赖性质谱采集技术(DIA-MS)因其无损的数据采集方式、高重复性和定量准确性等优点,在蛋白质组学研究中得到广泛应用^[31]^。然而,由于DIA-MS采集的肽段母离子和碎片离子之间的关联信息缺失,通常需要构建一个肽段谱图库,以便解析DIA数据^[31,32]^。肽段的多维分离是构建深度肽段谱图库的关键步骤。Ruedi Aebersold课题组^[32]^和Guo课题组^[33]^先后报道了适用于人蛋白质组分析的肽段谱图库,即泛人类谱库(PHL)和DIA泛人类谱库(DPHL)。其中用于构建PHL的331个样本中,14个样本经过了强阴离子交换(strong anion exchange, SAX)-RPLC离线二维分离,库中包含146576条肽段信息^[32]^。用于构建DPHL的1096个样本中,59个样本经过了SCX-RPLC离线二维分离,库中包含242476条肽段信息^[33]^。此外,Guo课题组^[34]^还报道了一个针对甲状腺肿瘤的肽段谱图库。研究者采集了来自不同类型甲状腺肿瘤组织的46个肽段样本,其中6个样本采用SCX-RPLC离线二维分离,40个样本进行了高/低pH RP-RPLC离线二维分离。尽管该研究的样本数量远低于前述两个泛人类肽段谱图库,但所有的样本经过充分的二维分离,最终肽段谱图库中包含了121960个肽段。

当肽段分离维度增加时,通常分离肽段所需的时间消耗也成倍增加。为了提高肽段分离的通量,本课题组^[35,36]^开发了二维毛细管阵列液相色谱系统用于高通量的肽段分离。其第一维分离采用IEC,第二维分离采用10或18根平行的RPC毛细管柱,这样从IEC洗脱的肽段可以在多根二维RPC柱上平行运行^[35,36]^。通过二维阵列分离系统,使色谱分离的速度提高10~18倍。

## 2 在自上而下的蛋白质组学中的应用

基因突变、多态性、RNA加工以及翻译后修饰如乙酰化、甲基化和磷酸化都可以导致单一基因产生多种功能上独特的“蛋白质变体(proteoforms)”^[37]^。TDP技术直接分析的对象是蛋白质,即通过质谱技术对完整蛋白质进行全面表征,这是全面表征蛋白质变体的最佳选择。当然,从技术角度看,TDP还存在一些复杂的问题需要解决,比如蛋白质的溶解性、蛋白质变体的复杂性,以及数据分析等问题^[38]^。基于HPLC的蛋白质分离因其自动化、高分辨且可以直接串联质谱等特点而广泛应用于TDP中蛋白质的分离。由于蛋白质平均分子质量远大于肽段,因此分离蛋白质时,固定相填料的孔径增加至25 nm或者更高(见表1)。

**表1:** 用于完整蛋白质分离的色谱模式及色谱柱填料参数

Analytes	Separation mode	Pore size/nm	Particle size/μm	Refs.
Rat or human liver protein lysate	SCX	30	5	[39,40]
	RPLC	30	5	
Human plasma	SAX	100	10	[41]
	RPLC	30	5	
HeLa or *E. coli* cell protein lysate	high-pH RPLC	30	3.5	[42,43]
	low-pH RPLC	30	5	
Caco-2 cell protein lysate	high-pH RPLC	100	8	[44]
	low-pH RPLC	30	3.5	
MCF10A cell protein lysate	SCX	30	5	[45]
	RPLC	/	5	
Myoglobin	SEC	30	2.7	[46]
Bovine serum albumin, thyroglobulin, pyruvate kinas, *γ*-globulin, trastuzumab, L-asparaginase, transferrin, ovalbumin, RNase A, uracil	SEC	25	4	[47]

SCX: strong-cation exchange; SAX: strong anion exchange; SEC: size-exclusion chromatography.

为了提高蛋白质鉴定的深度,多维色谱串联也是常用的方法。本课题组^[39⇓-41,48]^研发了一系列基于多维色谱的蛋白质分离策略,并成功应用于去除血浆或肝脏组织中的中高丰度蛋白质。我们通过二维SCX-RPLC分离系统成功地从大鼠肝脏组织样品中去除了77种中高丰度蛋白质^[39]^,在人肝脏组织样品中去除了58种中高丰度蛋白质^[40]^。此外,我们还通过SAX-RPLC二维分离系统成功去除了人血浆中的68种中高丰度蛋白质^[41]^。后续,我们开发的SAX-RPLC二维分离阵列进一步提高了二维分离的效率,一次实验中可去除84种血浆高丰度蛋白质^[48]^。通过多维分离降低生物样本中蛋白质的复杂度能够有效提高TDP的蛋白质鉴定通量。蛋白质多维分离策略包括且不限于HPLC、毛细管电泳、凝胶电泳等方案的组合,最近的一篇综述文章^[49]^详细讨论了这些不同分离技术组合在TDP研究中的应用。

### 2.1 蛋白质变性状态下的TDP

在TDP中,RPLC-MS同样是蛋白质鉴定最常用的方法^[37]^。需要注意的是,RPLC中高浓度有机溶剂通常会使蛋白质变性,因此不适用于有蛋白质结构解析要求的TDP^[50]^。与肽段分离相似,高/低pH RP-RPLC分离方案同样适用于TDP^[42,43]^。高/低pH RP-RPLC分离的正交性受到了平均肽段长度的限制,因为较短的肽段在不同pH下的带电分布有限^[45]^。如前文1.1节所述^[21]^,一个提高正交性的做法就是通过间隔合并不同时间点的馏分进行离线二维色谱分离以提高正交性。这样的做法虽然有效,但是却提高了整个分析流程的工作量,并且很难实现在线的二维分离。相比之下,完整蛋白质比肽段长得多,并且在不同pH下包含更多的带电基团,从而具有内在的高正交性。因此,高/低pH RP-RPLC二维分离模式可以用于完整蛋白质在线分离分析^[43]^。

Wu实验室^[42]^通过离线的高/低pH RP-RPLC平台分离细胞裂解完整蛋白质,验证了在蛋白质水平上的正交性。与使用一维分离时从124种蛋白质中鉴定出的225种蛋白质变体相比,使用高/低pH RP-RPLC二维分离方法鉴定了628种蛋白质中的2778种蛋白质变体^[42]^。接着,该团队又开发了在线二维高/低pH RP-RPLC系统。其工作原理是在第一维分离中使用高pH流动相,在第二维分离中使用低pH流动相稀释来自第一维分离的洗脱液并进行第二维分离。该自动化平台实现了超高的灵敏度,能够对5 μg样本中的1000多种完整蛋白质进行表征(图2)^[43]^。与一维RPLC相比,高/低pH RP-RPLC平台实现了良好的正交性、更高的分离效率和更全面的蛋白质表征。最近,Kaulich等^[44]^提出了一种新颖的低/低pH RP-RPLC方法,该研究采用了不同的离子配对试剂和固定相来改变两个RPLC维度之间的选择性^[44]^。第一维采用三氟乙酸作为离子对试剂,并使用聚合物反相材料(PLRP-S)作为固定相,而第二维采用甲酸作为离子对试剂,C4作为固定相。该研究的数据显示,通过改变离子对和固定相组成的二维RP-RPLC方案展示了比高/低pH RP-RPLC方案更强的分离能力(鉴定的蛋白质变体分别为4688和1788)^[44]^。然而,因为三氟乙酸与质谱不兼容,因此该二维液相色谱系统无法直接与质谱联用。

**图2:**
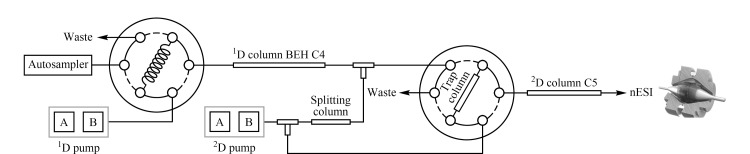
基于RPLC的在线二维分离示意图^[43]^

Brown等^[51]^报道了使用SEC-RPLC对内源性膜蛋白进行分离的TDP研究。由于在内源性膜蛋白提取过程中使用到表面活性剂,因此第一维的SEC也同时具有去除表面活性剂的功能。从第一维SEC洗脱的馏分经手动的体积浓缩之后通过RPLC-MS进行第二维的分离和蛋白质鉴定。Gomes等^[52]^通过SEC对牛精浆蛋白进行第一维分离之后分别通过毛细管区带电泳-质谱(CZE-MS)和RPLC-MS对牛精浆中的蛋白质变体进行了表征。CZE-MS和RPLC-MS分别鉴定了417和3090种蛋白质变体,CZE-MS低样本量限制(RPLC-MS的1/100)可能是导致二者鉴定量差异的主要原因。近期的一些研究也报道了IEC-RPLC用于TDP的在线二维分离方案。在一项腺相关病毒表征的研究^[53]^中,根据腺相关病毒封装DNA之后等电点发生差异的特点,研究者在第一维使用SAX来分离空衣壳和满衣壳的腺相关病毒。通过中心切割将第一维目标峰中的腺相关病毒转移至捕集柱,在捕集柱中完成腺相关病毒的蛋白变性和除盐,然后使用RPLC-MS对腺相关病毒的蛋白质进行第二维分离和鉴定。

### 2.2 蛋白质非变性状态下的TDP

蛋白质在非变性状态下直接进入质谱检测,可以获得独特的结构信息,包括一些不稳定蛋白质翻译后修饰位点的表征、金属结合位点、与小分子的非共价相互作用以及蛋白质的三级和四级结构等^[37]^。在这种方案中,因为流动相中含有的高浓度有机溶剂会使蛋白质变性,从而限制了该方法的应用。IEX、HIC和SEC是非变性蛋白质TDP方案中的主要色谱分离手段。它们的流动相主要包含一些磷酸盐、硫酸盐或柠檬酸盐,具有很好的生物兼容性。然而,这些非挥发性盐的存在使得在质谱分析时出现离子抑制和仪器污染,从而造成与ESI的不兼容^[54]^。一个可行的替代方案是采用挥发性盐(例如乙酸铵、甲酸铵或碳酸氢铵)作为流动相^[55]^,以提高质谱的兼容性。

研究者通过SEC-ESI-MS评估了挥发性盐对蛋白质结构的影响,实验结果显示,在一定的离子强度(0.01~0.2 mol/L)和pH(5.9~7.5)条件下,乙酸铵能有效保留蛋白质结构,而甲酸盐和碳酸氢盐会导致更高比例的蛋白质变性^[46]^。以挥发性盐为流动相,样本随溶剂进入离子源的时候通常需要高温蒸发掉溶剂,而这通常会破坏蛋白质的结构^[56]^。降低色谱流速可以减少进入离子源的溶剂含量,从而降低挥发溶剂所需的温度,这样的做法能降低蛋白质结构破坏的可能性^[47]^。研究人员通过减少SEC柱内径的方式实现了低流速,进一步优化了以挥发性盐为流动相的SEC-ESI-MS方案^[47]^。研究者使用了当前已商品化的内径为1 mm的SEC色谱柱,以实现流速15 μL/min的SEC-ESI-MS系统的分离检测。尽管较小的色谱柱可以提高灵敏度,但上样量受到严格的限制。在微升流速色谱条件下,增加样品体积可能导致谱带显著展宽,降低SEC的性能。SEC由于其非交互性质,无法通过在柱头捕集蛋白质来消除谱带展宽。因此,研究人员在SEC之前加入IEC捕集柱用于解决上样量受限的问题。使用低浓度盐(10 mmol/L或100 mmol/L乙酸铵)在低流速下将样品加载到IEC色谱柱中,然后使用高浓度的乙酸铵(400 mmol/L)进行洗脱,同时作为IEC的流动相。通过在IEC捕集柱上聚焦蛋白质并结合低流速SEC对蛋白质进行分离的方式,使蛋白质检出限低至200 ng^[47]^。

## 3 在蛋白-蛋白相互作用研究中的应用

蛋白-蛋白相互作用(protein-protein interaction, PPI)是指两个或多个蛋白质分子之间通过静电力、氢键、疏水作用等相互作用建立的高度特异性物理接触。这些特异性物理接触使蛋白质分子组成分子机器,执行各种生理功能,这些生理相互作用构成了生物体相互作用组学的核心^[57]^。蛋白质复合物(protein complex)是一类由非共价蛋白-蛋白相互作用连接在一起的分子机器,代表了蛋白质的四级结构的一种形式,这些复合物通常是生物过程的基本组成部分,细胞通常被视为由模块化的超分子复合物组成,每个复合物都执行着独立且离散的生物功能^[58]^。蛋白质复合物种类复杂,根据CORUM数据库的数据,目前已经发现超过4000种不同类型的蛋白质复合物^[59]^。因此,在对蛋白质复合物进行鉴定之前通常需要进行充分的分离。蛋白质组学在蛋白质复合物的分离和鉴定方面通常涉及以下4个主要步骤:(1)蛋白质复合物的提取;(2)蛋白质复合物的分离和馏分收集;(3)对每个馏分中的蛋白质进行鉴定和定量;(4)数据解析^[60]^。由于蛋白质复合物依赖非共价相互作用组装在一起,因此一旦蛋白质的活性受到外界因素(如温度、辐射、蛋白质变性剂等)的影响,蛋白质复合物的结构可能会发生变化。这一特性提示了蛋白质复合物提取和分离条件的温和性至关重要,以避免实验条件对蛋白质复合物的破坏。在蛋白质复合物提取过程中,通常采用高浓度的盐^[61]^或温和的表面活性剂(如DDM^[62,63]^、NP40^[64,65]^、TritonX-100^[66]^)来维持蛋白质复合物的稳定性。以盐为流动相的分离方法生物兼容性较好,通常是分离蛋白质复合物的首选方式。

### 3.1 基于SEC的蛋白质复合物的分离方法

与完整蛋白质相比,蛋白质复合物的显著特点之一是其具有较大的分子质量。根据UniProt (https://www.uniprot.org/)和CORUM数据库收录的人蛋白质和蛋白质复合物数据,蛋白质和蛋白质复合物分子质量中位数分别为46 kDa和207 kDa(图3a)。在分离蛋白质复合物时固定相填料的孔径进一步增加至50~100 nm^[65,67]^。SEC依据被分离物质分子质量的大小对其进行分离,其流动相通常是磷酸盐缓冲液或水,几乎不会对蛋白质复合物的结构造成破坏,因此,SEC广泛应用于蛋白质复合物的分离分析中^[61,65,68]^。

**图3:**
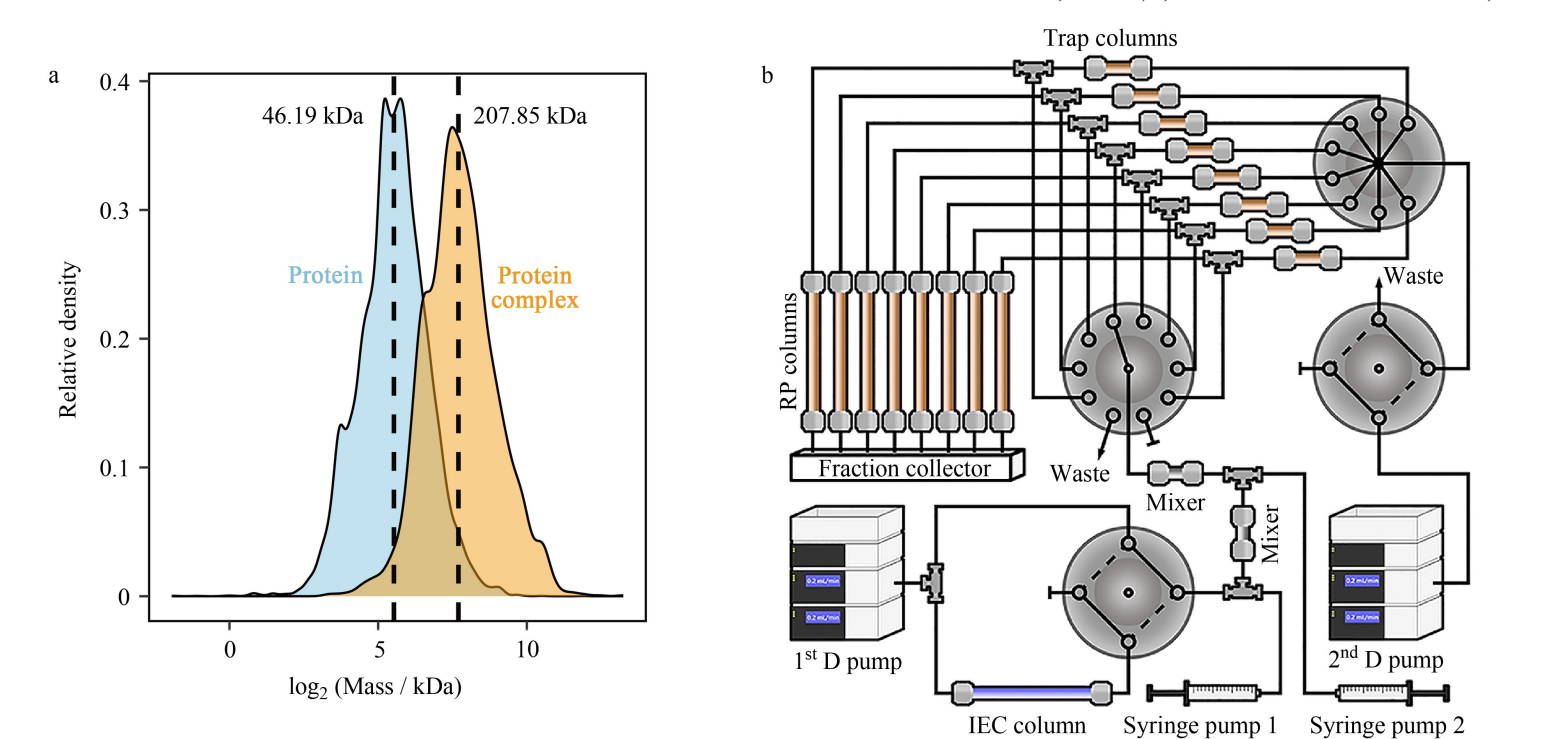
蛋白质复合物分离方案

Ruedi课题组^[65]^在应用SEC分离蛋白质复合物样本时引入了分子质量校正的方法。该研究通过SEC分析了5种分子质量不同的标准蛋白质。通过这些标准蛋白质的分子质量和在SEC中的色谱峰保留时间建立了线性关系,从而可以确定每个馏分分离出的物质的分子质量。由于SEC本身分辨率较低,每个SEC馏分可能包含数百到数千个蛋白质,其中大多数非相互作用蛋白质可能显示出难以区分的洗脱曲线。为了解决这一问题,该课题组分别开发了CCprofiler^[65]^和SECAT^[68]^算法,用于蛋白质复合物的鉴定和定量分析。

### 3.2 基于IEC的蛋白质复合物的分离方法

Emili课题组^[67]^系统报道了基于IEC-MS分析获得的人类可溶性蛋白质复合物图谱。该研究使用来自HeLa细胞的细胞质和细胞核蛋白质复合物提取物,分析了1163个生化分离的馏分(其中927个基于IEC分离),并系统地鉴定了由13993个高置信度蛋白质组成的网络,报告了622个推断的蛋白质复合物^[67]^。此外,该团队将蛋白质复合物的研究扩展到其他模式生物中,在9个不同物种中获得了6387个复合物馏分(其中5678个基于IEC分离)^[69]^。通过跨物种的蛋白质复合物鉴定,该研究首次报道了在物种进化过程中981个保守蛋白质复合物,以及蛋白质复合物的组成、系统发育分布和不同物种间的表型意义的独特差异。为了提高蛋白质复合物鉴定的通量,最近,该研究团队将经IEC分离的复合物馏分进行酶解,并使用六通路串联质量标签(tandem mass tag, TMT)进行标记。这一策略将基于IEC-MS的蛋白质复合物鉴定通量提高了6倍^[70]^。

### 3.3 基于IEC-RPLC的蛋白质复合物多维分离

RPLC在分离蛋白质和肽段方面具有卓越的分辨率,但其强疏水作用以及高有机相的流动相通常会破坏蛋白质复合物的结构。为了克服这个问题,本课题组^[66]^报道了采用甲醛交联来稳定蛋白质复合物的结构,然后进行RPLC分离的策略。这一策略首先通过评估对两个标准蛋白质复合物(Cam FERM Complex: *M*_W_=44 kDa, *K*_D_=5 μmol/L; IgG anti-IgG Complex: *M*_W_=290 kDa, *K*_D_=1 nmol/L)的分离来验证。研究结果表明,使用RPLC分离后,通过基质辅助激光解吸电离-质谱(MALDI-MS)和非变性凝胶电泳的验证,甲醛交联的蛋白质复合物能够保持其原始的完整结构。为了获得深度的蛋白质复合物图谱,研究^[66]^报道了基于IEC-RPLC的二维复合物在线分离方案。该方法将来自第一维IEC洗脱的馏分通过捕集柱分割到由8个反相柱组成的RPLC阵列中,并结合自制的多通道馏分收集器^[71]^,实现了在线的蛋白质复合物二维分离与收集(图3b)。通过深度分离,该研究从HeLa细胞中收集了256个蛋白质复合物馏分,并鉴定了1530个蛋白质复合物。

## 4 HPLC-MS应用拓展

### 4.1 在外泌体分析中的应用

外泌体(exosomes)是一种细胞外囊泡(extracellular vesicles, EVs),具有双层磷脂膜结构,包含蛋白质、RNA、脂类等多种物质的微小颗粒,其尺寸通常为30~150 nm。外泌体在细胞生长、增殖,尤其是肿瘤细胞的增殖和转移等方面发挥着关键的调节作用^[72]^。超速离心技术是外泌体提取分离最常用和报道最多的技术之一,也是外泌体提取分离的“金标准”。据估计,在外泌体研究中,超速离心技术占所有外泌体分离技术的50%以上^[73,74]^。由于外泌体与其他生物组分(如蛋白质和代谢物)存在明显的尺寸差异,因此SEC也常用于生物样本中外泌体的分离。SEC的分离条件相对温和,外泌体可以在等渗缓冲液中维持其生物学活性和完整的结构。将超速离心或超滤浓缩与SEC结合使用,可以获得纯度更高的外泌体,相较于单独使用超速离心或超滤浓缩,这一方法获得更佳的纯度^[75]^。

本课题组^[76]^采用超大孔径(200 nm)的体积排阻色谱柱,对尿源性外泌体进行多个亚组的高效分离,根据颗粒大小,分为大颗粒外泌体(>70 nm)、中颗粒外泌体(50~70 nm)和小颗粒外泌体(30~50 nm)。通过蛋白质组学分析发现,不同尺寸的外泌体亚组在蛋白质组成上存在显著差异,亚蛋白质组的主要蛋白质在功能分类和信号通路方面也表现出差异,包括蛋白质、核酸、糖和脂质代谢功能以及对细胞存活途径和细胞功能调节等方面的显著差异。这一研究为外泌体亚组的深入研究提供了有力的工具。

### 4.2 在单细胞分析中的应用

细胞异质性能够揭示同一个体中不同部分的情况,而常规生物学分析手段往往是将大量细胞混合在一起,最后得到的检测结果是大量细胞的平均结果,掩盖了细胞的异质性。同一组织中,不同细胞承担了不同的生理功能;同一个细胞在不同时期也会表现出不同的状态。因此能够获得带有空间、时间等信息的单细胞分析技术对推动生命科学的研究具有积极意义^[77]^。根据研究对象不同,单细胞组学分析包括单细胞基因组分析^[78]^、单细胞转录组分析^[79]^、单细胞蛋白质组分析^[80]^、单细胞代谢组分析^[81]^以及表观基因组分析^[82]^等。就单细胞蛋白质组而言,目前质谱仪器的灵敏度足以用于分析单细胞内低丰度的蛋白质,部分仪器的检出限达到10^-18^mol^[83]^,结合HPLC的分离能力,基于HPLC-MS的方法具有分析单个细胞整个蛋白质组的潜力。

单细胞蛋白质组分析的难点主要是样品中的蛋白质含量太少,哺乳动物体细胞内的蛋白质含量仅为0.1 ng^[84]^,而使用鸟枪法进行蛋白质组学分析时,细胞需要经过一系列复杂的处理才能够进入质谱分析。目标物在样品处理过程中以及后续色谱分析时极易被吸附,造成严重损失。同时,因样品量太少,实验过程对检测系统的灵敏度要求极高。因此需要建立合适的样品处理方法,尽量减少损失。

传统大体积蛋白质组样品的处理方式并不适用于少量细胞的研究。研究者们对于少量细胞的样品处理方式进行了尝试^[85⇓⇓⇓-89]^。对于需要进入质谱分析的单细胞样品,它的处理过程要求更为苛刻。大部分方法在操作时使用含有表面活性剂和其他化学物质的缓冲溶液来制备样品。而这些试剂往往和质谱不兼容,需要使用超滤或者置换等方式来进行除盐除杂。因此不可避免会造成样品损失,而这样的损失对于单细胞分析来说可能是致命的。因此,单细胞蛋白质组分析中,样品处理非常关键。开发出一个不引入质谱不兼容物质且能够尽量减小样品接触面积的方法,对于提高最后鉴定到的蛋白质数量具有非常重要的意义。

Shi课题组^[90]^提出了简便的表面活性剂辅助的一锅样品前处理-质谱法(SOPs-MS),并对50~1000个MCF10A细胞中的1200~2700个蛋白质进行定量,样品处理体积为50 μL,能够兼容目前使用的商业化实验器械。但是对于单细胞样品来说,μL级体积的接触面积仍然很大,减小样品制备的体积可以减小样品与外界接触的表面积,从而减小吸附损失。因此也有研究者将关注点放在减小样品的处理体积上。

本课题组^[91]^在2015年开发的iPAD-100(an integrated proteome analysis device for 100 cells)系统通过一次性将细胞与裂解试剂、酶的混合液共同吸入毛细管中,然后在系统中进行消化、富集,直接分离分析,实现了对于100个杜克氏C型结直肠腺癌(DLD-1)细胞的分析。在细胞被引入系统后,程序能自动运行,避免复杂的样品处理步骤,减少样品损失,防止样品污染,提高灵敏度。100个细胞处理的体积为3.2 μL,最后在100个DLD细胞中鉴定到651种蛋白质。之后,我们在iPAD-100的基础上进行改进,于2018年提出了iPAD-1(an integrated proteome analysis device for 1 cells)方法^[92]^。直接将2 nL含有单个细胞、裂解试剂和酶的混合液吸入22 μm的毛细管中进行超声、加热处理,并使用22 μm内径的短毛细管柱分析,一个样品的分析时间为1 h,最后在1个HeLa细胞内成功鉴定到181种蛋白质。

Fang课题组^[93]^在2013年提出了一种用于pL级液体加样的全自动系统(SODA)。系统使用锥形毛细管作为操作管,使用注射泵来控制注射针连接毛细管液面移动。液滴的操作在一个玻璃基底的微芯片上进行,表面有2 mm厚的油层防止液体蒸发。通过编写好的程序,毛细管逐步进行液体的吸取和滴加,油层的密封使得pL级的液滴基本没有蒸发损失。2018年,Zhu等^[94]^报道了一套NanoPOTS(nanodroplet processing in one pot for trace samples)系统,实现了对10~100个哺乳动物细胞的蛋白质组分析,成功在10个细胞中检测到1500种蛋白质。NanoPOTS将玻璃芯片作为液体操作的平台,使用盖子减少反应过程中的液体蒸发。为减小接触面积,芯片上的样品点为亲水表面,而周围进行疏水处理,样品因为表面张力的作用可以只与凸起的表面接触,减小接触面积,最后处理的液滴体积为200 nL。2020年,该课题组^[95]^实现了nanoPOTs系统与LC-MS的连接,能够自动化地进行上样和分析。通过在系统中加入十通阀进行切换,实现冻干的酶解液复溶及液相色谱-质谱检测。此系统,在每天处理24个细胞的情况下,平均鉴定到256种蛋白质。

## 5 结语

基于HPLC-MS的蛋白质组学已然成为当前生物化学领域对蛋白质全面表征与定量的工具,高效的色谱分离是深度蛋白质组鉴定的必要条件。在BUP中,对极微量肽段样本分离并匹配当前高灵敏度的质谱检测是多维色谱分离肽段的发展趋势。采用在线的多维分离方案或者使用微升流速以及纳升流速级色谱系统都是有潜力的方法。在TDP中,选择色谱模式时要考虑色谱流动相与被分离样本的兼容性。对于一些有活性要求的分离对象如需要维持构象的蛋白质,通常要避免使用含有高浓度有机相的分离模式,避免破坏蛋白质活性,同时流动相也要兼顾与质谱的兼容性。如果分离对象可以直接进入MS分析,就能在提高检测通量的同时减少额外的样本处理导致的样本损失。在蛋白质组学领域,近年来涌现出的单细胞蛋白质组学对色谱工作者提出了更高的要求。目前微量样本前处理技术已经成熟,接下来如何将色谱分离系统与微量样本前处理系统有效结合以减少样本转移的损失需要进一步的研究与探索。色谱技术的不断创新和改进将在未来的蛋白质组学研究中发挥关键作用,为生物医学领域的进展提供更强大的工具。

## 作者团队简介

现代色谱分离分析研究组隶属于复旦大学化学系,在张祥民、高明霞两位教授带领下,研究组从2000年以来一直致力于蛋白质组学新技术、新方法的研究,发展多维色谱分离以及生物质谱鉴定新技术,研制色谱仪器及其关键技术。承担了多项国家级科研项目,与国内外相关研究组建立了良好合作关系。

课题组网站: https://hplc.fudan.edu.cn/。

**Figure:**
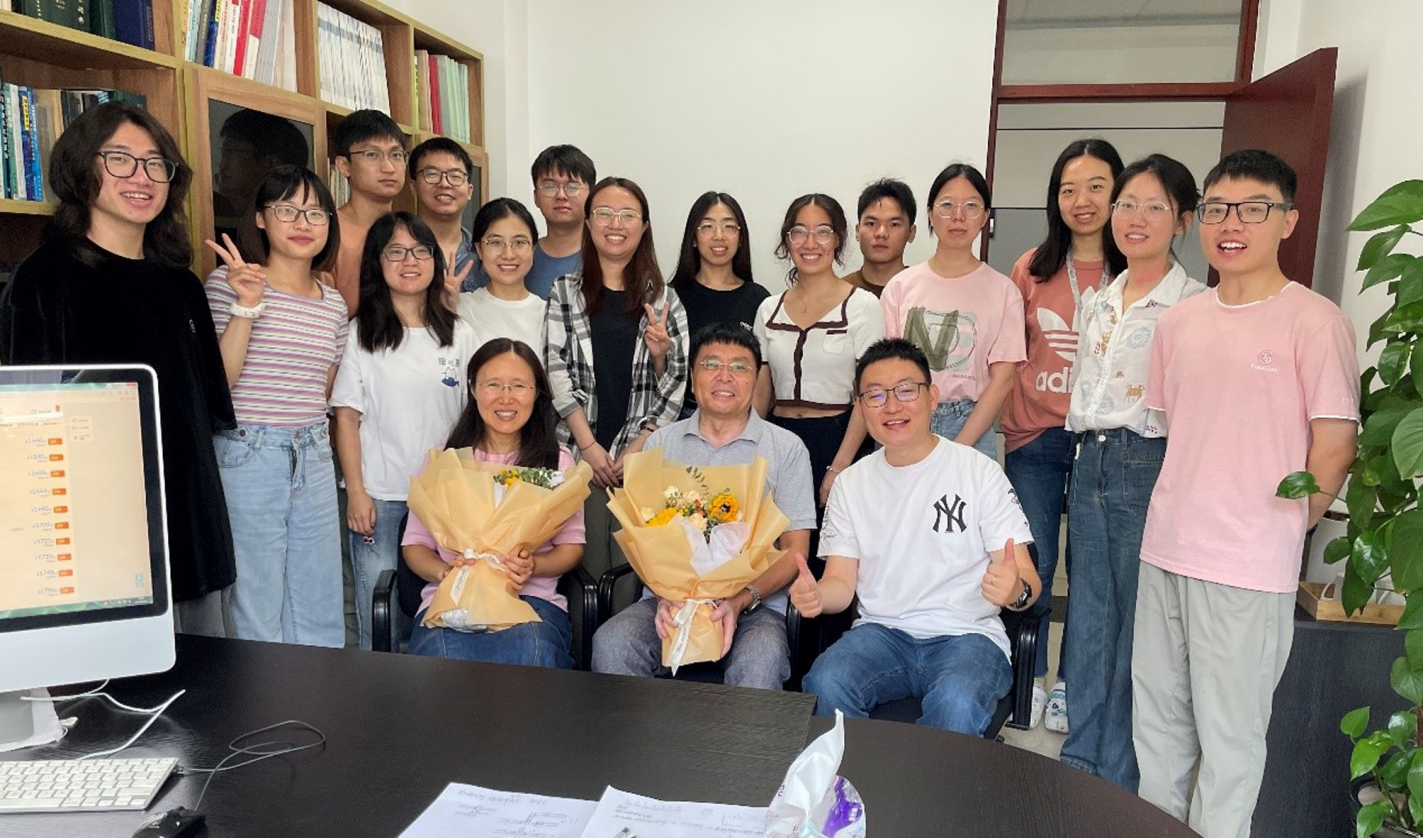


### 人才队伍

**研究组组长:**张祥民教授

**研究组成员及学生:**教授2人,副教授1人,高级工程师1人,博士后1人,研究生十余人

**团队精神:**凝心聚力,致力于发展高效色谱分离、高灵敏度质谱鉴定新方法

### 科研项目及成果

**科研项目:**国家“973”计划、“863”计划、重点研发计划项目,国家自然科学基金仪器专项、面上项目,上海市科技攻关项目等数十项

**科研成果:**建立液相色谱热膨胀微流泵、阵列多维液相色谱、单细胞蛋白质组分析平台,外泌体、CTC细胞的捕获与分析平台等;在*Angew Chem Int Ed*, *Adv Mater*, *Anal Chem*, *J Proteome Res*, *Proteomics*, *J Chromatogr A*等期刊发表论文300余篇;申请国家发明专利近百项;培养研究生、博士后研究人员近百人

**获奖情况:**教育部自然科学一等奖(2006),“上海市优秀研究生论文”指导教师奖(2003,2009),优秀研究生导师称号(2008),复旦大学“全国百篇优秀博士论文”导师提名奖(2011),教育部自然科学二等奖(2012),中国烟草总公司科技进步二等奖(2019),化学会第24届全国色谱学术报告会“中国色谱贡献奖”(2023)等

### 研究领域

**Figure:**